# Authors’ Response to Peer Reviews of “Perception and Impact of White Spot Lesions in Young People Undergoing Orthodontic Treatment and Their Guardians: Protocol for a Mixed Methods Study”

**DOI:** 10.2196/80139

**Published:** 2025-09-12

**Authors:** Amaar Obaid Hassan, Janine Doughty, Jayne Harrison

**Affiliations:** 1 Department of Orthodontics, School of Dentistry, Liverpool University, Pembroke Place, Liverpool, L3 5PS, United Kingdom; 2 School of Dentistry, University of Liverpool, Liverpool, United Kingdom; 3 Orthodontic Department, Liverpool University Dental Hospital, Liverpool, United Kingdom

**Keywords:** orthodontics, white spot lesions, fixed appliances, dentistry

*This is the authors’ response to peer-review reports for “Perception and Impact of White Spot Lesions in Young People Undergoing Orthodontic Treatment and Their Guardians: Protocol for a Mixed Methods Study.”*

## Round 1 Review

### Reviewer A [1]

#### General Comments

*This paper [2] appears to me to be well-written and adequately cited. I believe that this paper will contribute to the literature once the study commences and the data are collected and analyzed. However, I do have some questions/concerns regarding the study design and potential data analysis that I have included in my comments below. I would like the authors of this paper to review these comments/recommendations and to either implement them as they see fit or justify why they believe they do not need to.*

#### Specific Comments

##### Major Comments

*B1. Line 170, you state that this study is purely descriptive, so a power analysis is not required. How will you control for confounding variables such as cultural beliefs which may be over- or underrepresented in your participant pool? Additionally, how will you ensure that your participant demographics allow for the generalization of this paper’s findings to patient populations outside of Liverpool?*

**Response:** Following the advice of the peer review, we have undertaken a pilot study including 20 people, and this has enabled us to complete a power calculation and sample size, meaning we are able to provide statistically significant data of the representative sample. However, if we want a representative sample from patients receiving routine orthodontic treatment in our department, with 11 providers and about 50 patients each, this gives a population of 550 and with a 95% confidence level and error margin of 5%, we would need 226 respondents.

*B2. Line 203, you mention that sampling will be based on age, gender, ethnicity, etc. However, Table 2 does not mention ethnicity. Could you edit Table 2 to mention ethnicity or edit Line 203 to remove ethnicity. I would recommend editing the table because I believe the participant demographics to be important, especially since different cultures may approach esthetics and health beliefs differently. This concern regarding culture connects with major comment 1.*

**Response:** The table has been amended to add ethnicity, meaning that at least 4 of 12 participants will be required to be from a minority ethnic background, thank you.

##### Minor Comments

*B3. Line 129, “Sponsorship will be sort from...,” please change to “Sponsorship will be sought from...”*

**Response:** As now approved, we have changed this to: “Sponsorship has been obtained from Liverpool University Hospitals NHS Foundation Trust (UoL001871).”

*B4. Line 167, you state that a sample size of 200 respondents is sufficient for Part 1 of the study. Could you justify this estimate in a more thorough way other than stating that it is a “pragmatic estimation?”*

**Response:** We have undertaken a sample size calculation following a pilot study where we invited 20 participants to provide feedback on the questionnaire. Using this data, we were able to complete an analysis of the statistical difference and thus justify the sample size using a power calculation. See above response to B1.

*B5. Line 173, you state that participants will be contacted on the same day as their orthodontic appointment. Will this be before or after the appointment? Will participants be compensated for their time? How will you ensure that participants’ rights are respected and that they do not feel pressured into participating?*

**Response:** Participants will be compensated for their time, and the statement “They will have free choice over whether they wish to take part and will be able to read the participant information leaflet during their appointment or after. Should they wish to take part, then they will be reimbursed for their time, with a £10 electronic voucher for the questionnaire, and £25 for the qualitative research” has been added. In the pilot, 25% of people declined to take part even with an offer of an electronic voucher as reimbursement. We have amended the timeline following the pilot study and have increased the time for recruitment to 12 months, meaning there will be ample time for recruitment and no pressure for the research team. Participants will be contacted while they attend their orthodontic appointment.

*B6. Line 427, “or childs name?” please change to “or child’s name?”*

**Response:** We are unable to identify the error you have stated.

### Reviewer AH [3]

*C1. The abstract of this paper must be revised. “Several studies explore the prevention and/or treatment of WSL” is inappropriate for the abstract.*

**Response:** We have amended this to “Although there have been studies that have investigated the prevention and treatment for WSL, there remain uncertainties about what young people and their parents or guardians know or feel about them.”

*C2. The Methods section describes the mixed methods approach well, but the recruitment process could be elaborated. For example, how will convenience sampling be conducted to avoid bias? Including qualitative and quantitative data is well-justified, but there is no mention of how the two datasets will be integrated into the analysis. More details on how the qualitative data will expand upon the quantitative findings would strengthen the methodology.*

**Response:** We have agreed to review the data collection after 25% of data collection has been completed. We will check age, gender, ethnicity, stage of treatment, Index of Orthodontic Treatment Need, and condition of first molar teeth and determine whether we feel this is consistent with the representative sample of the population. If it is not, we will target more underrepresented groups. One hundred is a large sample size, and many of our patients have not commenced with orthodontic treatment or are above the age of 15 and do not meet our inclusion criteria, meaning that those who do meet the inclusion criteria are likely to be contacted to take part. We have added the following statement: “The authors will review the data after 25% completion of the quantitative study to check if participants who have been recruited fit the demographics of the clinic. If any people are underrepresented at this point, then this will be identified, and more efforts will be made to recruit people from the underrepresented groups.” We know from a previous audit the people who have brace treatment at the clinic are representative of the sample population for deprivation status. We have included details on how the data will be integrated, stating that “The mixed methods study design is to provide enriched data by augmenting the quantitative findings with qualitative interviews. An explanatory sequential mixed methods approach will be used, whereby the qualitative data will expand on the understanding gained from the questionnaire [4]. The diagram below (Figure 2) illustrates the different parts of the study and at what point the mixing of the data will occur. The quantitative and qualitative parts of the research will be analyzed for convergent and divergent data interpretation of the mixed methods research that compares both datasets. Figure 2. Flowchart of mixed methods design.” We have also included a flowchart (see Figure 2), illustrating how the data are to be integrated. For clarity, we have also added the following statements: “Following completion of the quantitative research, we will organize meetings with the research team and patient and public involvement group to review the data and develop the interview schedule based on the findings of the first part of the research” and “Following completion of the data collection/analysis of both datasets, the results will be merged. The quantitative and qualitative data will then be compared for convergence and divergence.”

*C3. The sample size rationale is explained well, though stating why a power analysis is unnecessary for a descriptive study could prevent confusion.*

**Response:** Since we have received feedback for the study, we have undertaken a power analysis and sample size calculation based on a pilot study we completed. See the response to B1.

*C4. In the Results section, it would be useful to clarify how data from questionnaires and interviews will be compared and whether there is an expectation of divergence between parent and child responses.*

**Response:** We are currently undertaking a statistical analysis on questions in the questionnaire between parents and children so that we are able to compare answers between children and parents. Questions we are looking at comparing include “before braces and after braces are removed but with WSL” photos and “how likely you think you will get WSL.” We are unsure if there will be an expectation of divergence between parent and child responses; although in the pilot, we gathered information from 10 children and 10 parents/guardians, and these answers did differ (parents expected that their child was more likely to get white spot lesions [WSLs] and were more unhappy about getting WSLs compared to the children). The overall κ statistic for these questions was 0.284 (95% CI 0.029-0.539), with individual questions ranging from −0.152 to 0.98, suggesting that there was fair agreement but that there was considerable variation. This will be explored further and discussed once the study has been completed. We are expecting that the qualitative research will have similar findings to the quantitative part. We have added the following statement in the Results section: “We will also use the κ statistic to determine whether there are differences between parents’/guardians’ and young people’s answers to the questions in the questionnaire.”

*C5. The Limitations section acknowledges some important aspects, such as recruiting from only one hospital, but it does not address potential biases in self-reported data. There is also no mention of how the study will address participants’ potential reluctance to report negative experiences due to social desirability bias. Expanding on these limitations and how the study will mitigate them would improve transparency.*

**Response:** We think that we will address potential biases in self-reported data by using statistical analysis in the quantitative research to help confirm study findings, and we will also use a coding framework (NVivo) to generate codes/themes (rather than developing codes ourselves) to try to limit self-reported bias. We have also included a researcher in the team who is not a clinician to assist with recruitment and data collection and analysis. We have commented in the study that multiple people are interpreting codes/data. We have used a patient and public involvement group to develop the research and ensure that it is patient-focused, and we will continue to do this to develop a questionnaire and interview schedule to avoid leading questions. We have written up the study protocol, which has undergone peer review as part of the grant application and ethical approval processes. This will help to ensure transparency and has involved a diverse group of opinions to identify potential sources of bias. We have also published our questionnaire and interview schedule, which has been added as an appendix. With qualitative research, there is always a limitation of self-reported bias; however, we have attempted to limit this. To clarify this in the Limitations section, we have identified the following statements: “Although one of the limitations of survey and qualitative research includes the potential risk of self-reporting bias, the authors have attempted to address this by publishing the study protocol and using patient and public involvement to develop the research and help to analyze/interpret the data. The authors will publish the protocol, the questionnaire/interview schedule, and data so that readers are able to make an informed decision about the potential sources of bias. Data analysis will be reviewed by a researcher who is a nonclinician and NVivo will be used to limit self-reporting and ensure a systematic framework to coding” and “Participants also have the opportunity to review study findings to ensure that they agree with the results” and “During the qualitative research analysis, data coding and themes of transcripts will be undertaken by AOH using NVivo 12. The transcripts and codes/themes generated will be sent to a second or third researcher to confirm reliability (JH, JD, or AR).” We have attempted to address social desirability bias by asking the young participants not to discuss answers with parents/guardians as this may influence their answers. Participants are advised that the reason for separate questionnaires is so that they can answer their questions honestly and that the authors are able to compare answers. The participants can complete the questionnaire in a private room without a researcher being present. The study has used patient and public involvement throughout to ensure questions are relevant to the participants and not misleading. The following statements have been added to the Limitations section: “The authors have also attempted to address self-reporting bias by publishing the study protocol, the questionnaire/interview schedule, and data so that readers are able to make an informed decision about the potential sources of bias” and “Social desirability bias has been limited by asking participants not to discuss answers with parents/guardians as this may influence their answers. The participants are able complete the questionnaire in a private room without a researcher being present. The study will not recruit any participants who are under the clinical care of the research team involved in recruiting. Patient and public involvement will be used throughout all stages of the research to ensure questions are relevant to the participants and not misleading.”

*C6. The potential psychological impact of WSLs could be expanded upon in the Discussion, especially regarding how WSLs may affect patient compliance and satisfaction posttreatment.*

**Response:** Negative association following WSLs has already been discussed in the Discussion section. We have added the following statement regarding improving compliance for preventing WSLs in the Discussion section: “Even with effective oral hygiene instruction, around half of young people do not follow the clinician’s advice to improve their oral hygiene [5]. The COM-B model is presented as a tool to diagnose which of capability, opportunity, or motivation need to change for a new behaviour to take place [6]. Although interventions designed to improve oral hygiene during orthodontic treatment (including using smartphones, a toothbrushing app, visual aids, motivational interviewing, oral health reinforcements) have been looked at, it is only the use of mobile phones that have limited evidence for improving oral health during orthodontic treatment [7]. To our knowledge, trials/studies have not been undertaken to explore barriers to oral hygiene or behavioural interventions to reduce WSL formation during orthodontic treatment in young people.”

*C7. To expand the Discussion, the following article must be cited: Jamloo H, Majidi K, Noroozian N, et al. Effect of fluoride on preventing orthodontics treatments-induced white spot lesions: an umbrella meta-analysis. Clin Investig Orthod. April 19, 2024;83(2):53‐60. [doi: 10.1080/27705781.2024.2342732]*

**Response:** We have added the reference in the Introduction section.

## Abbreviations

WSL: white spot lesion
